# Visual evoked potentials to change in coloration of a moving bar

**DOI:** 10.3389/fnhum.2014.00019

**Published:** 2014-01-24

**Authors:** Carolina Murd, Kairi Kreegipuu, Nele Kuldkepp, Aire Raidvee, Maria Tamm, Jüri Allik

**Affiliations:** ^1^Institute of Psychology, University of Tartu, TartuEstonia; ^2^Doctoral School of Behavioural, Social and Health Sciences, University of Tartu, TartuEstonia; ^3^Institute of Public Law, University of Tartu, TallinnEstonia; ^4^Estonian Academy of SciencesEstonia

**Keywords:** motion, velocity, color change, reaction time, visual evoked potentials

## Abstract

In our previous study we found that it takes less time to detect coloration change in a moving object compared to coloration change in a stationary one (Kreegipuu etal., 2006). Here, we replicated the experiment, but in addition to reaction times (RTs) we measured visual evoked potentials (VEPs), to see whether this effect of motion is revealed at the cortical level of information processing. We asked our subjects to detect changes in coloration of stationary (0^°^/s) and moving bars (4.4 and 17.6^°^/s). Psychophysical results replicate the findings from the previous study showing decreased RTs to coloration changes with increase of velocity of the color changing stimulus. The effect of velocity on VEPs was opposite to the one found on RTs. Except for component N1, the amplitudes of VEPs elicited by the coloration change of faster moving objects were reduced than those elicited by the coloration change of slower moving or stationary objects. The only significant effect of velocity on latency of peaks was found for P2 in frontal region. The results are discussed in the light of change-to-change interval and the two methods reflecting different processing mechanisms.

## INTRODUCTION

The perception of motion is one of evolutionary oldest abilities of the visual system. As it enables us to cope with a dynamic environment, it seems reasonable to assume that the presence of motion information is not easily ignored even when attending to another quality of an object, like its form or color.

Researchers have identified at least two distinct functional subsystems, one of which processes color (parvocellular pathway) and the other motion (magnocellular pathway). The subpopulations of these pathways are evident in retina, projecting through LGN to V1 (Hubel and Wiesel, 1972; Livingstone and Hubel, 1988). From V1 the information is transmitted through ventral and dorsal streams (Goodale and Milner, 1992). The dorsal stream (also referred to as “where”/”how” pathway) gets its input mostly from the magnocellular pathway and projects to posterior parietal lobe. The dorsal stream has been most commonly associated with awareness of object location and guidance of action. The ventral stream (the “what” pathway) gets both magno- and parvocellular input and projects to temporal lobe. This stream has been associated with attention, object recognition and identification. The dorsal stream has been considered to be relatively faster than the ventral stream (Norman, 2002), but it has also been suggested that these two streams are highly interactive (Dobkins and Albright, 1993; Cicerone et al., 1995). These two distinct subsystems are additional evidence of the evolutionary pressure for development of a system specialized for early detection of motion.

The aforementioned visual streams involve specialized areas in the cortex that are activated when processing color (“globs” in V4 and adjacent areas, see Conway et al., 2007) and motion (MT/V5, Zeki, 1974). V5 has been shown to react to luminance changes of an object, but it is not activated by isoluminant, heterochromatic stimuli (Conway et al., 2007). Differently from luminance contrast sensitivity, the magnocellular layers in LGN have not been demonstrated to be color selective. The processing of motion information has been believed to be rather unaffected by color (in some stages of the processing), however, it has been suggested that some magnocellular neurons respond to chromatic contrast, but without concrete information about its sign (Dobkins and Albright, 1993). The color processing mechanisms on different stages get their input from both magno- and parvocellular pathways (e.g., double-opponent cells and thin stripes in V2; Gegenfurtner, 2003; Shapley and Hawken, 2011). Taken together, it is clear that parvo- and magnocellular subsystems interact with each other (Dobkins and Albright, 1993; Cicerone et al., 1995; for a review see Skottun, 2013), and therefore the characteristics of one quality can influence the perception of the other (Moller and Hurlbert, 1997; Kreegipuu et al., 2006; Werner, 2007).

It has been suggested by the different latencies theory that stimulus qualities (like color, luminance, shape, motion) have different processing latencies, and the processing latency for color precedes processing of motion by 70–80 ms (Moutoussis and Zeki, 1997). However, by now many studies have indicated that the visual delays for different visual attributes are neither fixed nor identical, but rather depend on different stimulus characteristics, as well as on the experimental set up (Allik and Kreegipuu, 1998; Gauch and Kerzel, 2008).

Kreegipuu et al. (2006) conducted a simple reaction time (RT) study where subjects were asked to detect the color or luminance change of moving or stationary stimuli. The results showed shorter RTs to color or luminance change for faster moving stimuli compared to more slowly moving or stationary stimuli. However, this unexpected discovery that it takes more time to notice change in color of a stationary object rather than of the same object put in motion – was not generalizable to all types of motion. We observed shorter detection times only with a single moving object, not with moving gratings covering an extended portion of the visual field (Murd et al., 2009). It seems that an identifiable object traveling along a solitary trajectory is critical for improved ability to detect change in coloration.

There is an agreement between researchers that Reichardt-type motion energy detectors are the main building blocks of many motion analysing mechanisms (Reichardt, 1961; Poggio and Reichardt, 1973; Van Santen and Sperling, 1985). However, beside motion energy, motion can be recovered based on some higher-order perceptual attributes. For example, according to one conceptualization it is possible to distinguish three motion detection systems at least: a first-order system that uses a primitive motion energy computation to extract motion from moving luminance modulations; a second-order system that uses motion energy to extract motion from moving texture-contrast modulations; and a third-order system that tracks features (Van Santen and Sperling, 1985). It seems that the observed pattern – the effect of velocity appearing only with single moving objects (Kreegipuu et al., 2006) but not with large moving gratings (Murd et al., 2009) – fits nicely to this theoretical scheme. The question remains whether this advantage of a single moving stimulus, when compared to a stationary coloration-changing stimulus, appears already on the cortical level of information processing. One approach to address this question is to measure the brain’s electrical activity by electroencephalography (EEG) and compare the transient visual evoked potentials (VEPs) of the coloration change between different stimulus conditions (stationary, slow, and fast moving stimuli). This would enable us to see whether the stimulus condition effects the evoked potentials of coloration change causing amplitude and/or latency differences in some components, such as N1, P2, N2, and P3.

Based on the literature on event-related potentials (ERPs; Fonaryova Key et al., 2005; Luck, 2005; McKeefry, 2001), there are some results indicating we might find a difference in VEPs between color-change events in stationary versus moving stimuli. For example, McKeefry (2001) found that amplitudes of positive components P1 and P2 and negative component N2 for the motion onset of chromatic stimuli were reduced for slow moving stimuli than for fast moving stimuli. Since this tendency was not present when motion onset of luminance stimuli for two velocities was compared, it was concluded that this effect of velocity found for the onset of chromatic stimuli might indicate shifting between two separate mechanisms – parvocellular and magnocellular. According to this theory, parvocellular mechanism is active with slow moving chromatic stimuli and magnocellular mechanism with fast moving chromatic stimuli. Therefore, when comparing VEPs of color change in fast and slow moving stimuli, we might find reduced amplitudes in slower moving stimulus.

It has also been suggested that the visual N1 reflects the discrimination process within the focus of attention (Vogel and Luck, 2000). Some studies of selective attention and cueing have shown that N1 amplitude to attended (and validly cued) stimuli is larger (more negative; Luck et al., 1994). Beer and Röder (2004) have suggested that attention to motion enhances processing of visual stimuli, since N1 amplitudes for stimuli moving in the attended direction were more negative compared to stimuli moving in the unattended direction.

As the task in our previous study (Kreegipuu et al., 2006) required a quick response, it presumed directing attention to the stimulus. Since the characteristics of a moving stimulus enable both spatial and temporal predictions about the event, there might be somewhat different expectations about the coloration-change of a moving stimulus compared to the stationary stimulus. Taken into account the previous findings, there seems to be enough reason to consider that this advantage of a moving stimulus will be seen on the cortical level of information processing.

## MATERIALS AND METHODS

### PARTICIPANTS

Seven participants (six females and one male, aged 20–25) took part in this experiment. One of the subjects was well-trained; other six were naïve concerning the specific purposes of this study. Participants were informed about the general purpose of the experiment (comparison of the data gathered by using psychophysical and electrophysiological methods) and given an overiew of the equipment used in the experiment. Participants were also informed about their right to quit the experiment any time they wished, and gave their informed consent. All participants self-reported to have normal or corrected-to-normal vision and reported no deficits in color perception.

### STIMULI

A rectangular bar with luminosity profile corresponding to the positive half-cycle of a sine wave (1.2 × 2.3° at 90 cm viewing distance) was presented as a stimulus on the screen of a Mitshubishi Diamond Pro 2070SB monitor (frame rate 140 Hz; 752 × 564 pxl; 27.6 × 20.5° at 90 cm viewing distance). The bar was either red (CIE chromaticity coordinates: 0.636; 0.335) or green (CIE chromaticity coordinates: 0.289; 0.607) with luminance of 13.85 cd/m^2^, luminance was measured at the peak of the positive phase of the sinusoidal luminance profile. The neutral uniform background of the screen had a luminance of 0.3 cd/m^2^. A white fixation point (8 × 8 minof arc) was present on the screen for the entire trial. Stimulus was rendered with Cambridge *ViSaGe* visual stimulus generator (Cambridge Research Systems Ltd., Rochester, UK). As the red and green color were photometrically isoluminant and we did not measure subjective isoluminance (and the colors were not therefore corrected on these basis), we use the term “coloration change” – as an arrangement of color and tones – to be more precise as the color change might have been subjectively accompanied by small luminance artifacts.

### PROCEDURE

Each trial started with the appearance of a moving or stationary test stimulus. The moving stimulus appeared at the left or right edge of the screen and started to move horizontally across the screen with a velocity of 4.4° or 17.6°/s.

**FIGURE 1:**
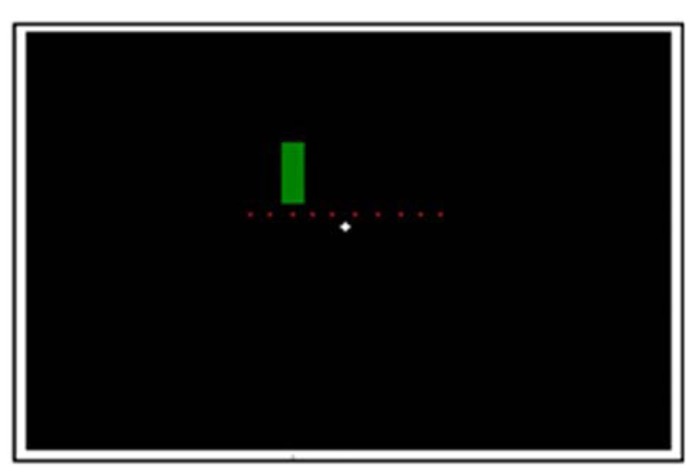
**Experimental setup**. The dots indicate the 10 possible color switch-points in the middle 1/3 of the screen (not shown on the actual screen).

**Figure 1** demonstrates the experimental setup. In each trial, coloration change (from red to green or vice versa) took place in one of ten possible switch points in the middle third of the screen (equally spaced positions: 9.2°; 10.22°; 11.24°; 12.26°; 13.28°; 14.3°; 15.32°; 16.34°; 17.36°; 18.38° from the starting edge). The stationary stimulus (from here on also referred to as velocity 0°/s) appeared randomly in one of these ten positions and changed its coloration unpredictably in a time window of 476–3547 ms after its appearance (which in average corresponds to the coloration change of a stimulus moving with velocity of 10°/s). Time windows for the coloration change of moving stimuli were: 480–885 ms after its appearance for a faster moving stimulus and 1929–3547 ms for a slower moving stimulus.

Subjects were instructed to press a response button as quickly as possible after the detection of a change in coloration. RTs were saved for offline analyses. Each observer performed two blocks of 150 trials, in total 300 trials – 100 per velocity condition (0°, 4.4, 17.6°/s). The order of trials with different velocities was pseudo-randomized within the experimental block and there was a pause of 3 s (inter-stimulus interval) before the beginning of each trial. When a response was not given, the missed trial was repeated on random position in the experimental block.

### ELECTROENCEPHALOGRAPHY

The electroencephalogram (EEG) was registered with BioSemi’s system Active One (*BioSemi*, Amsterdam, The Netherlands), and Vision Analyzer 1.05 (Brain Products, GmbH, Munich, Germany) was used for offline data analysis. 14 active electrodes (Fz, Fpz, F3, F4, P3, P4, C3, C4, Cz, Pz, T5, T6, O1, O2) were used according to the international 10/20 system electrode placement (Jasper, 1958), off-line referenced to ears. Additionally, the Common Mode Sense (CMS) active electrode was placed between Fz and Cz and the Driven Right Leg (DRL) passive electrode on the observer’s neck. Vertical and horizontal eye movements were registered with two bipolar electrodes for both. The DC mode and sample rate of 1024 Hz was applied for online recording. Data were offline filtered (0.3 Hz low cut-off and 35 Hz high cut-off filters, both 24 dB/oct) and epoched around the coloration change event (-100 to +500 ms). Ocular artefacts were removed with the built-in Gratton and Coles algorithm (Gratton et al., 1983) used by Vision Analyzer that corrects ocular artefacts by subtracting the voltages of the eye channels, multiplied by a channel-dependent correction factor, from respective EEG channels.

A 100 ms interval before the coloration-change was selected for baseline correction and segments were tested for several known artefacts (50 μV allowed voltage step per sampling point, maximal allowed difference within the segment 100 μV, maximal absolute amplitude ± 70 μV and lowest activity criterion of 0.5 μV per 100 ms). Segments were averaged for different velocities and observers. Automatic peak detection (separate search for every channel) for local maximum/minimum was used to find ERP component peaks for N1 (50–130 ms), P2 (130–170 ms), N2 (150–270 ms) and P3 (230–500 ms). Time intervals for peak detection were set based on the grand average data and visually inspected to be suitable for all subjects. Since the visual inspection did not reveal any overlapping contrapolar peaks, the electrodes were pooled as follows: frontal (Fz, Fpz, F3, F4), parietal (P3, P4, Pz), central (C3, C4, Cz), temporal (T5, T6), occipital (O1, O2).

Repeated measures analysis of variance (ANOVA; Statistica 10.0, StatSoft Inc., Tulsa, OK, USA) was used for analysis of both RTs and VEPs.

## RESULTS

### Reaction Times

**FIGURE 2:**
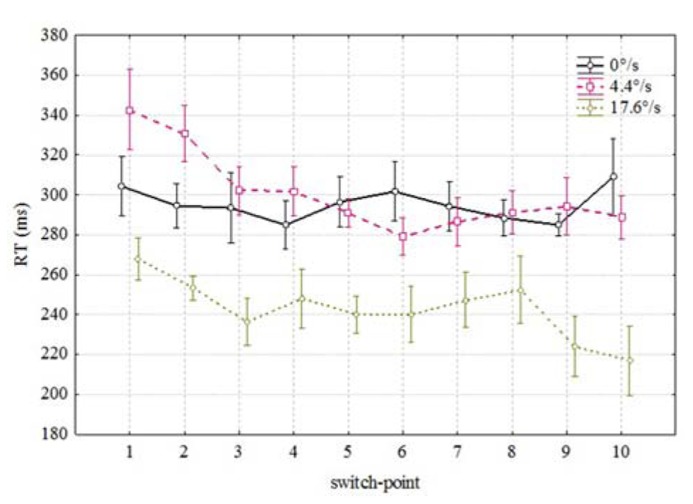
**Mean RTs as a function of spatial position of the color change along the movement trajectory**. Vertical bars denote ± standard error.

**Figure 2** shows the averaged RTs in each 10 possible coloration-switch points for three velocities of the moving bar: 0 (stationary), 4.4, and 17.6°/s. RTs over 1000 ms and below 100 ms were excluded from the analysis. Over all subjects, there were 16 misses (RT > 1000 ms) and 146 anticipated responses (RT < 100 ms) out of 2100 responses.

Since there was no effect of direction (stimulus moving from right to left or vice versa) detected on the RTs [*F*(1,3) = 3.141, *p* < 0.1745] we omitted this parameter from the further analysis. **Figure 2** reveals two conspicuous properties. First, it seems to take less time to notice the coloration change which happens during the later portion of the movement trajectory [*F*(9,54) = 3.39, *p* < 0.002]. As can be seen from **Figure 2**, mean RTs were shorter for coloration changes occurring in the last positions (correlation between RT and switch-point *r* = -0.056 *p* < 0.01). Second, it took considerably less time to notice the coloration change of a fast moving (17.6/s) bar than the coloration change of the same bar moving slowly (4.4°/s) or standing in the same position [*F*(2,12) = 71.52, *p* < 0.00001]. Thus, it seems to be confirmed that mean RTs to the coloration change of the faster moving stimulus were shorter than in case of the slower moving or stationary stimulus. There was also an interaction between velocity and switch-point position [*F*(18,108) = 1.7, *p* < 0.051] which indicates that the order of RTs at different positions is not identical.

### VISUAL EVOKED POTENTIALS

**FIGURE 3:**
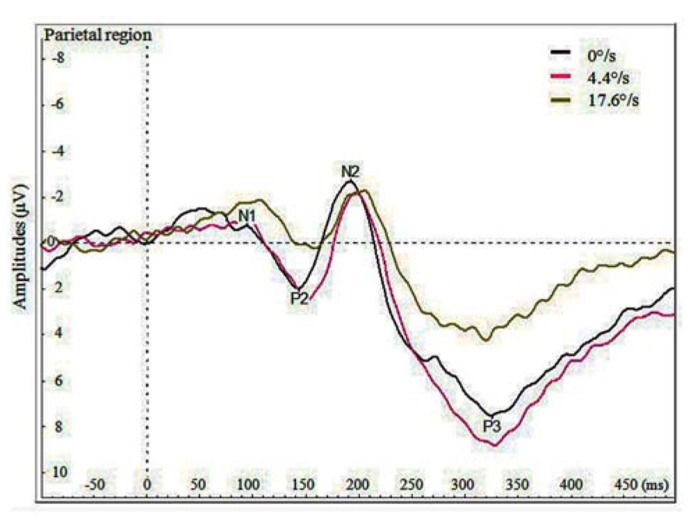
**Average VEPs for the color change in the parietal region by three velocities (0, 4.4, and 17.6°/s)**.

**Figure 3** demonstrates the grand average potentials in parietal region where the components were most pronounced. The figure presents data pooled together over the data of seven participants for the three velocities. Like manual RTs, VEPs elicited by the coloration change of the fast moving stimulus (17.6°/s) are different by both amplitude and delay compared to those elicited by the coloration change of the slow moving and stationary stimulus. Repeated measures ANOVA was conducted on mean peak amplitudes of pooled regions of interest (listed at the end of Method section). The significant effect of velocity on N1 amplitude was found in frontal [*F*(2,12) = 4.464, *p* < 0.036] and in central region [*F*(1,12) = 4.501, *p* < 0.035]. This effect demonstrates a difference between N1 amplitudes for the coloration change of slower and faster moving stimuli, showing larger amplitudes in case of faster moving stimuli. Although five out of seven participant also showed similar tendency in parietal region, the overall effect remained insignificant [*F*(2,12) = 2.382, *p* < 0.135]. Significant effect of velocity on P2 amplitude was found in frontal [*F*(2,12) = 8.41, *p* < 0.0053], central [*F*(2,12) = 12.92, *p* < 0.0011] and parietal region [*F*(2,12) = 19.775, *p* < 0.0002], showing less pronounced amplitudes for faster versus slowly moving stimuli.

Significant effect of velocity on N2 amplitude was found in frontal [*F*(2,12) = 8.41, *p* < 0.0052] and central region [*F*(2,12) = 12.92, *p* < 0.0011], showing larger N2 with slower moving stimuli. Significant effect of velocity was also found on P3 amplitude in central [*F*(2,12) = 5.068, *p* < 0.0254] and parietal region [*F*(2,12) = 10.814, *p* < 0.0021], showing stronger P3 amplitudes for the coloration-change of slower moving and stationary stimuli.

The only significant effect of velocity on latency of peaks was found for P2 in frontal region [*F*(2,12) = 6.359, *p* < 0.014], so that the peak was earliest for the coloration change of the stationary stimulus.

Surprisingly, as is shown in **Figure 3** and by the statistics presented, the amplitudes of P2, N2, and P3 components were reduced for the coloration change of the faster moving stimulus. In frontal and central regions, we did find the amplitude of component N1 to be significantly larger (i.e. more negative) for the coloration change of the faster moving stimulus, but the N1 amplitudes for slower moving and stationary stimulus did not differ significantly.

However, the amplitudes of P2 and P3 seem to be lined up according to the average of the time windows of coloration change – as we described in the Method section, the stationary stimulus changed its coloration 476–3547 ms (corresponding in average to coloration change of a bar moving with velocity of 10°/s), the faster moving stimulus 480–885 ms and the slower moving stimulus 1929–3547 ms after the beginning of the trial.

**FIGURE 4:**
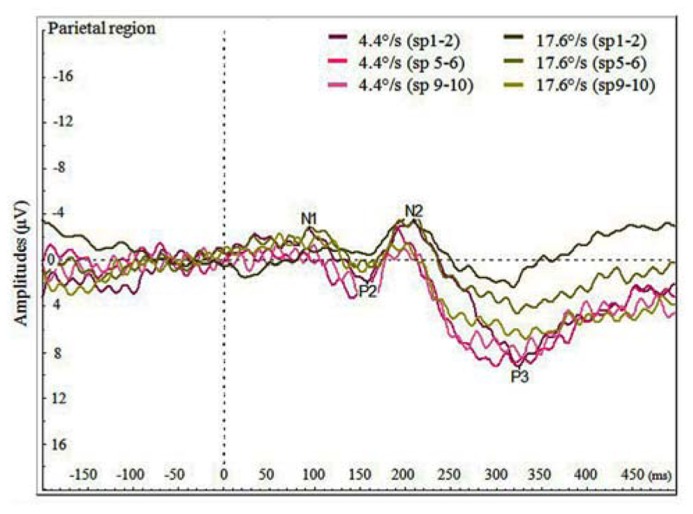
**Average VEPs for the color change in the parietal region by faster and slower moving stimuli (4.4 and 17.6°/s) for pooled switch-points of the color change (first two switch-points sp1–2, two middle switch-points sp5–6 and last two switch-points sp9–10)**.

**FIGURE 5:**
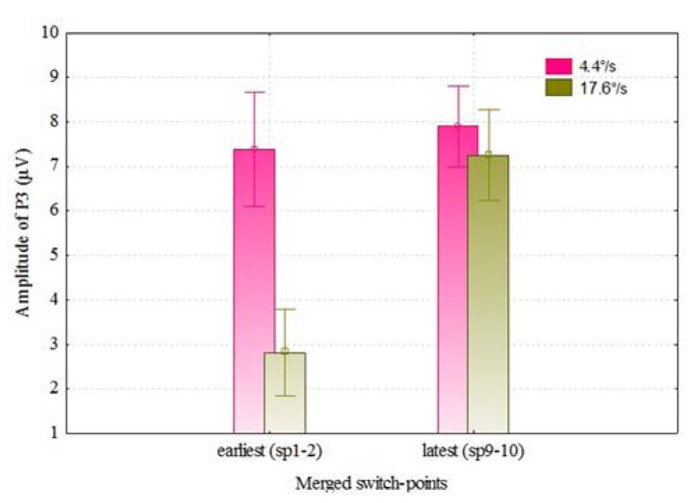
**P3 amplitudes (over F, C, T, P, and O regions) for the color change of slower and faster moving stimuli by two earliest (sp1–2) and latest switch-points (sp9–10)**. Vertical bars denote ± standard error.

We also analyzed the VEPs by the switch-points of coloration change (see **Figure 4**), and noticed that with faster moving stimulus the amplitude of P3 increased with later switch-points, but this trend was not present with slower moving stimuli. In **Figure 5**, P3 amplitude by the merged coloration-change switch-points (two earliest versus two latest on the motion trajectory) are presented.

### CHANGE-TO-CHANGE INTERVAL ANALYSIS

**FIGURE 6:**
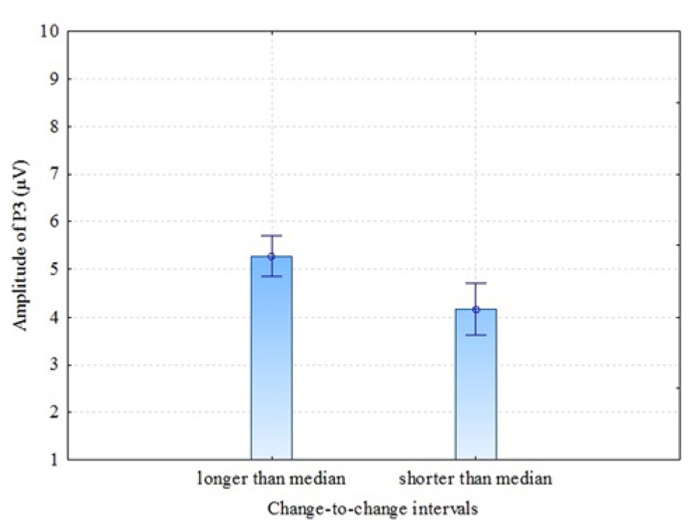
**P3 amplitudes (parietal region) by longer and shorter than median change-to-change intervals (TTI)**. Vertical bars denote ± standard error.

There are some previous studies (Gonsalvez et al., 2007; Gonsalvez and Polich, 2002) that have found previous-target-to-next-target interval (TTI) to have an effect on P3 amplitude: the amplitude is larger when the TTI is longer. In our experiment, conditions were presented in random order (not in blocks of velocity) and the time between coloration change in one trial and the next trial varied. Therefore, it was interesting to test whether or not our results of P3 amplitude in parietal electrodes (where P3 was most pronounced) demonstrate TTI – in our case coloration-change-to-coloration-change – effect. This interval is a sum of (a) the time from one coloration change until the end of the present trial, (b) the time between trials (which was 3 s in our experiment) and (c) the time from the beginning of the next trial until the coloration change of this trial. For analysis we divided change-to-change intervals into two: change-to-change intervals longer than the median and change-to-change intervals shorter than the median. The individual medians of change-to-change interval varied between 6.7 and 7.1 seconds (as a result of the randomly varied time window of the coloration change of the stationary stimulus). The comparison was made between these two groups for P3 amplitude in pooled parietal region. The results were as follows: dependent samples *t*-test *t* = 3.63 (df = 6; *p* = 0.011), Cohen’s *d* = 1.37, showing that longer than median change-to-change interval trials had considerably larger P3 amplitude compared to shorter than median change-to-change interval trials (see **Figure 6**). It looks like the next VEP elicited by the change of coloration was of higher amplitude when more time had passed from the coloration-change in the previous trial. These results confirm Gonsalvez and Polich (2002) observation that TTI is a critical variable in P3 response.

Mean RTs, divided into two groups by the same principle as for VEPs, did not show statistically significant effect of TTI: dependent samples *t*-test *t* = 2.405 (df = 6; *p* = 0.053).

RT and TTI were correlated by velocity condition (0°/s, 4.4°/s, 17.6°/s), the correlations were insignificant for the stationary stimulus (0°/s) *r* = -0.04, *p* = 0.344 and faster moving stimulus (17.6°/s) *r* = -0.075, *p* = 0.061, but significant for slower moving stimulus (4.4°/s) *r* = -0.13, *p* = 0.001. Again, the response was attenuated for a faster moving stimulus.

When analysing only the trials with change-to-change interval covered by all velocities – interval from 5488 to 7617 ms –, the effect of velocity on mean RTs was still significant [*F*(2,12) = 58.68, *p* < 0.00001], which means that the main effect of velocity on RTs is independent of change-to-change interval.

## DISCUSSION

The behavioral results of our experiment were in a good agreement with our previous study (see Figure 2 in Kreegipuu et al., 2006) showing that the faster the speed of the moving stimulus is, the shorter is the time that is required to detect an instant change in its coloration. For some reason, it takes less time to notice the change in coloration of a relatively fast moving object than the coloration change that happens to the same object if it moves more slowly or stays at the same place. Like RTs, VEPs elicited by coloration-change seem to be able to distinguish between objects that remain stationary or move with different velocities. However, on average evoked potentials to coloration-change of the fast moving object were smaller and their maximal amplitude was reached with a longer delay when compared to evoked potentials to coloration-change of slow moving or stationary objects. Thus, RTs and VEP amplitudes were negatively correlated. For example, VEPs elicited by the coloration-change of the fast moving (17.6°/s) bar had smaller amplitude of P2 and N2 peaks and longer latency of the P2 peak than the peaks elicited by the coloration-change of slowly moving (4.4°/s) or stationary (0°/s) bars.

There are many studies showing reasonable agreement between psychophysical and electrophysiological results (Wolf et al., 1988; Donchin and Lindsley, 1966; Kreegipuu and Allik, 2007). For example, there was a considerable homology between the temporal structure of RTs and VEP intervals when the task was to detect onset or offset of motion (Kreegipuu and Allik, 2007). Both manual reactions and VEPs increase in latency as the velocity of the onset or offset motion decreases and are well approximated by the same negative power function with the exponent close to -2/3 (Dzhafarov et al., 1993; Kreegipuu and Allik, 2007). It is important to remember that in our current study velocity was not a critical attribute to attend. Participants were instructed to ignore motion and react, as fast as possible, to the first noticeable change in coloration of a uniformly moving or stationary bar. In principle, it was expected that the velocity of the test object has only minor effect on the ability to notice a sudden change in coloration. Nevertheless, we observed that the velocity of the test object exerted a considerable effect on both, RTs and VEPs. According to manual RTs, it took less time to notice the coloration-change of a fast moving object but according to VEPs, this change elicited smaller deflections from the base level which were also delayed in time.

One mechanism that could cause the reduction of VEP amplitude at relatively high velocities is lateral or temporal masking (Sperling, 1965). When an object moves rapidly, a place where coloration-change happened will be flanked by a nearby place to which the moving object has reached a few moments later. The VEP signal generated by the stimulus activity in this new place may interfere with the signal elicited by the stimulus in the previous position. Since these two similar signals are out of phase, their summary activity is expected to be reduced in amplitude compared to their amplitudes in isolation. Unfortunately, our data are fragmented to tell exactly from which velocity this potential mechanism could become efficient. At the current moment we can only guess that this critical velocity must be somewhere between 5 and 17°/s.

Whatever the cause of the VEP amplitude suppression at higher velocities is, the discrepancy between manual RT and evoked potentials is puzzling. There is nothing new in the finding that RT data sometimes disagree with VEP results. Although many studies have shown good agreement between evoked potentials and psychophysical data, there are quite a few studies showing discrepancy between these two measures (Crognale et al., 1997; McKerral et al., 2001; Chakor et al., 2005). Some of these disagreements could be caused by the magno- and parvocellular pathways’ specialized input to ventral and dorsal streams. The fact that the dorsal stream – that is presumably specialized for action – receives mostly magnocellular input.

One of the reviewers guided our attention to the circumstance that as subjective isoluminance of colors may not be in accordance with photometric isoluminance and may vary depending on the retinal eccentricity. It is possible that the chromatic change was accompanied by small luminance artifacts (as mentioned in the Method section). We have also shown in our previous study (Kreegipuu et al., 2006) that identical effect of velocity on RTs we have repeatedly found for color changes was also found for luminance changes. However, in this achromatic change condition the luminance changed from 5.09 to 20.2 cd/m^2^ (or vice versa). This is considerable luminance change and it is unlikely that the possible luminance artifacts accompanying chromatic change would solely be responsible for identical results. It has also been shown that even in presence of low values of luminance contrast, the chromatic information is highly relevant for detecting a stimulus (O’Donell et al., 2010).

Several studies have demonstrated that the color aberration and isoluminance value related to retinal eccentricity vary depending on the target extent and spatial frequency (Bilodeau and Faubert, 1997; Barboni et al., 2013). However, Bilodeau and Faubert (1997) have shown that while they manipulated with spatial frequency and size of the target, the isoluminance values within central 20 degrees did not change. Psychophysical data [which has been considered to be more sensitive to luminance changes than electrophysiological measurements (e.g. Rabin et al., 1994)] from our previous study (Murd et al., 2009) indicates that the chromatic aberration and/or luminance modulations related to retinal eccentricity do not explain the effect of velocity found on RTs when changes in coloration were detected. We found no difference in the effect of velocity on response times whether subjects were asked to keep central fixation or to follow the stimulus with a gaze (i.e. the location of the target on the retina did not change). Both conditions showed a similar significant effect of velocity on response times and this effect was present for all subjects (Murd et al., 2009).

It has been suggested that some magnocellular neurons signal temporal alternation between light of equal luminance, without signaling the sign of the chromatic contrast (Dobkins and Albright, 1993; Baker et al., 1998). In our display, motion was both chromatically and achromatically (as there was luminance difference between background and the stimulus) defined, and as the colors (red and green) were not presented simultaneously, it is hard to tell whether the transient color change could have been mediated by this unsigned chromatic contrast detecting mechanism or not. But if considering it as a possibility and taking into account the finding that the sensitivity of VEPs to parvo- and magnocellular input are different (Tobimatsu et al., 1995; Foxe et al., 2008), – so that VEPs are more pronounced for parvocellular input and might not always adequately reflect magnocellular inputs (see Foxe et al., 2008) – this would explain why simple RTs to the color change are more influenced by object’s velocity than VEPs.

Also, Di Russo and Spinelli (1999) showed in their study on the effect of spatial attention in chromatic and luminance stimuli, that VEPs did not reveal any latency differences between attended and unattended conditions when chromatic stimuli were used. They suggested that spatial attention is mainly controlled by visual areas considered to be part of the dorsal stream. Therefore, in the light of the abovementioned studies, the discrepancy between RT and VEP results might be explained by findings that these two measures reflect information processing in different streams (for similar results see also Highsmith and Crognale, 2010).

However, there is a considerable amount of critique regarding the extent of the independence of dorsal (action) and ventral (perception) systems and whether the specialization is relative rather than absolute (see the discussion paper by Schenk and McIntosh, 2010; also Himmelback et al., 2012). Sperandio and colleagues (Sperandio et al., 2010) demonstrated in visual illusion experiments that simple RTs – differently from other types of motor behavior (grasping) – are affected by the illusion, although it has been presumed that the dorsal stream is not sensitive to illusions. Their results showed that RT varied as a function of perceived (rather than physical) stimulus properties. Therefore, simple RT is likely to be an outcome of interconnection with the ventral stream. In general, this may mean that recorded VEP signatures are reflecting some neurophysiological mechanisms that are not identical to mechanisms which form the basis for manual RTs. Thus, manual reaction is elicited in this particular case by an internal representation which is not explicitly manifested in the recorded VEP signatures.

It is very unlikely that change-to-change interval has anything to do with the suppression of the VEP amplitude at higher velocities. However, the influence of target-to-target interval on the amplitude of P3 has been demonstrated in some previous studies with both auditory and visual stimuli (Gonsalvez et al., 1999,^2007^; Gonsalvez and Polich, 2002). Gonsalvez and Polich (2002) tested TTIs up to 16 seconds and found that when the TTI was relatively long, the P3 amplitudes remained constant, indicating that the increase of P3 amplitude with shorter TTIs might be explained by resource limitation or limitations on memory-updating operations. Since we conducted a simple single-task experiment (requiring no comparisons between targets and non-targets), the more probable explanation is that our results refer to the capacity of the visual system to “recover” from one event and to be ready for processing the next one. Therefore, it seems that for simple tasks that require a quick response, it is not crucial to have the total amount of resources available for the cortical processing.

To conclude, our results fall in line with the view that although human visual system may have functionally distinct information processing streams that receive their input from brain areas and pathways specialized on different stimulus characteristics, they are highly interactive in several levels. The question of where the results of psychophysical and EEG measurements meet and to what extent can they explain each other still needs some further investigation.

## AUTHOR CONTRIBUTIONS

Carolina Murd, Kairi Kreegipuu, and Jüri Allik formulated the research question. Aire Raidvee programmed the experimental setup. Carolina Murd, Nele Kuldkepp, and Maria Tamm collected the data. Carolina Murd and Kairi Kreegipuu analyzed the data. Carolina Murd drafted the manuscript and in cooperation with Kairi Kreegipuu, Jüri Allik, Nele Kuldkepp, Aire Raidvee, and Maria Tamm revised it to its final form.

## Conflict of Interest Statement

The authors declare that the research was conducted in the absence of any commercial or financial relationships that could be construed as a potential conflict of interest.
